# Dog ownership, dog walking, and leisure-time walking among Taiwanese metropolitan and nonmetropolitan older adults

**DOI:** 10.1186/s12877-018-0772-9

**Published:** 2018-04-04

**Authors:** Yung Liao, Pin-Hsuan Huang, Yi-Ling Chen, Ming-Chun Hsueh, Shao-Hsi Chang

**Affiliations:** 1 Department of Health Promotion and Health Education, National Taiwan Normal University, 162, Heping East Road Section 1, Taipei, 106 Taiwan; 2 Department of Physical Education, National Taiwan Normal University, Taipei, 106 Taiwan

**Keywords:** Walking, Dog ownership, Dog walking, Taiwanese, Older adult

## Abstract

**Background:**

This study examined the prevalence of dog ownership and dog walking and its association with leisure-time walking among metropolitan and nonmetropolitan older adults.

**Methods:**

A telephone-based cross-sectional survey targeting Taiwanese older adults was conducted in November 2016. Data related to dog ownership, time spent dog walking (categorized as non-dog owner, non-dog walkers, and dog walkers), and sociodemographic variables were obtained from 1074 older adults. Adjusted binary logistic regression was then performed.

**Results:**

In this sample, 12% of Taiwanese older adults owned a dog and 31% of them walked their dogs for an average of 232.13 min over 5.9 days/week (standard deviation = 2.03)**.** Older adults living in nonmetropolitan areas were more likely to own a dog (14.7% vs. 9.1%) but less likely to walk their dog (25.9% vs. 39.6%) than were those living in metropolitan areas. Compared with non-dog owners, only older adults living in nonmetropolitan areas who were dog walkers achieved 150 min of leisure-time walking (odds ratio: 3.03, 95% confidence interval: 1.05–8.77), after adjustment for potential confounders.

**Conclusion:**

Older Taiwanese adults living in nonmetropolitan areas who owned and walked their dogs were more likely to achieve health-enhancing levels of leisure-time walking. Tailored physical activity interventions for promoting dog walking should be developed for older adults who are dog owners living in nonmetropolitan areas and who do not engage in dog walking.

## Background

Physical inactivity is associated with increased risks of mortality and noncommunicable diseases, such as cardiovascular disease, type 2 diabetes, certain types of cancer, and dementia [1, 2]; it also poses a substantial economic burden [3]. In particular, the association between physical inactivity and health outcomes or functional independence is more evident in older adults because older adult populations are the least physically active age group [4]. Although many countries have a national physical activity policy or plan, Sallis et al. [2] reported that physical activity is not increasing worldwide, and that 23.8% of the global population remains physically inactive. In Taiwan, nearly 40% of Taiwanese older adults failed to achieve the minimum recommendations of physical activity [5]. Therefore, an urgent need exists to develop effective population-based intervention strategies that encourage Taiwanese older adults to engage in sufficient physical activity.

Dog ownership has been widely considered to be a potential method for increasing and maintaining physical activity levels [6, 7]. A growing body of literature has reported that older adults who are dog owners are more physically active than are non-dog owners or other pet owners [8, 9], because dog ownership increases behavioral intention through the dog’s positive effect on its owner’s cognitive beliefs about walking; moreover, dogs provide an essential form of social support that motivates dog owners to walk [10]. However, previous studies examining associations of dog ownership and dog-walking status with physical activity have stated that although dog ownership appears to facilitate walking behavior, only a portion of older dog owners walk their dogs (a range of 22%–77% has been reported by studies in the United Kingdom, the United States, and Japan); nevertheless, those who walked their dogs are more likely to meet the recommended levels of physical activity [8, 11–14]. Notably, most of these previous studies on older adults have been conducted in well-developed countries, such as the United Kingdom [11], the United States, [8, 12, 13], and Japan [14]; have focused on total physical activity; and have examined samples across the general adult to older adult populations. By contrast, little is known about the prevalence of dog ownership and dog walking and their associations with leisure-time walking among older adults in other cultures and environments, such as Taiwan.

Moreover, the pattern of dog-walking behavior differs between high-density and low-density cities (i.e., dog owners living in low-density cities may be less likely to walk their dog because they have larger yards or public open spaces for dogs to be active without needing to be walked) [14, 15]. Thus, the prevalence of dog ownership and dog-walking status might differ according to cities with different residential densities (i.e., metropolitan vs. nonmetropolitan areas), and this could be the potential moderator of the associations between dog ownership and walking status and leisure-time walking. However, few studies have examined these associations according to metropolitan and nonmetropolitan areas. Thus, this study first described the prevalence of dog ownership and dog-walking status in metropolitan and nonmetropolitan areas, and then examined the associations of dog ownership and walking status with leisure-time walking among older adults residing in metropolitan and nonmetropolitan areas.

## Methods

### Participants

This study used data from a cross-sectional telephone-based survey, conducted by a research service company using a computer-assisted interviewing system, in November 2016 in Taiwan. It was estimated to have an older adult population of 6,142,472 with an area of 36,192.8 km^2^ in Taiwan. The needed sample size for the present study was 1067 older adults, which was calculated using a 95% confidence level and a 3% confidence interval. A standardized questionnaire was administered by trained interviewers, who had experience in administering telephone population surveys and received 2 days of training before the start of each survey. The response rate was 30.3% (3546 older adults were contacted, and 1074 of them finished the survey). The telephone research service company did not offer any rewards for participation. Verbal informed consent was received before the beginning of each telephone interview. The protocols of this study were reviewed and approved by the Research Ethics Committee of National Taiwan Normal University (REC number: 201605HM006).

### Dog ownership and dog-walking status

Dog-related variables in the present study were dog ownership and dog-walking behavior. Participants were asked if they owned a dog (yes/no), and if so, whether they walked the dog (yes/no), the usual frequency of walks (days/weeks), and the duration of the walks (minutes/day). These validated items measuring dog-walking frequency and duration for older adults have been used in previous studies [13, 14]. Dog ownership and walking status were categorized (using dog ownership and time spent dog walking) into three groups: (a) non-dog owners, (b) non-dog walkers (defined as dog owners who did not walk their dog), and (c) dog walkers (defıned as dog owners who walked their dogs for at least 10 min/week) [13, 14].

### Leisure-time walking

Time spent in leisure-time walking was obtained using the Taiwanese version of the International Physical Activity Questionnaire-long version (IPAQ-LV) [16], which is widely used for telephone-based surveys among middle-to-older adults [17, 18]. IPAQ-LV had a high test–retest reliability (*r* = 0.78) and acceptable criterion validity (*r* = 0.31–0.41) in comparison with accelerometers [16]. Time spent in leisure-time walking was measured using the fourth part of the IPAQ-LV, and was calculated by multiplying the frequency (per week) by the duration of leisure time (per day). Because the distributions were skewed, the outcome variable was categorized on the basis of the IPAQ scoring protocol [19]. Furthermore, based on the physical activity recommendation for older adults [20], the outcome variable was dichotomized into “achieving 150 min/week” and “not achieving 150 min/week.”

### Sociodemographic variables

The sociodemographic variables included gender, age, type of occupation, educational level, marital status, living status, residential area, self-rated health status, and body mass index (BMI). Age was categorized into three categories: 65 to 74 years, 75 to 84 years, and older than 85 years. Occupational type was classified into “full-time job” and “not full-time job.” Educational level was divided into: “non-tertiary degree” (< 13 years) and “tertiary degree” (≥ 13 years). Marital status was categorized as “married” and “unmarried” (including widowed, separated, and divorced). Living status was divided into “living with others” and “living alone,” and residential area was categorized into “metropolitan” and “nonmetropolitan” areas. Self-rated health status was categorized into “good” and “poor,” and BMI score was based on self-reported weight and height and grouped into two categories: “not overweight” (< 24 kg/m^2^) and “overweight/obese” (≥ 24 kg/m^2^) according to Taiwanese cut-off points.

### Statistical analysis

The data of 1074 older adults who had complete information for the study variables were analyzed. Chi-squared tests were used to evaluate the proportional differences in sociodemographic variables and Mann-Whitney U tests were used for continuous variables between the residents of metropolitan and nonmetropolitan areas. In addition, forced-entry adjusted logistic regression was conducted to estimate the odds ratios (ORs) of achieving 150 min of leisure-time walking according to the categories of dog ownership and dog-walking status, after adjustment for gender and age (Model 1) and the other potential confounders (Model 2); notably, this analysis also examined all of the respondents as one group, and the respondents stratified by metropolitan or nonmetropolitan area. Adjusted ORs and 95% CIs were calculated for each variable. Inferential statistics were obtained using IBM SPSS 23.0, and the level of significance was set at *p* < 0.05.

## Results

### Sociodemographic characteristics of the participants

Table 1 contains the sociodemographic characteristics in the total sample according to metropolitan and nonmetropolitan areas. Overall, the mean (standard deviation [SD]) age of the respondents was 72.51 (± 6.2) years. 50.3% of respondents were men, 65.2% were aged 65–74 years, 28.5% had a tertiary degree, 10.1% had a full-time job, 76.8% were married, 86.4% were living with others, 20.0% of respondents reported health status were poor, and 41.8% were either overweight/obese. A total of 49.0% respondents lived in metropolitan areas. Chi-square test analysis revealed that older adults living in nonmetropolitan area were more likely to be male (54.7% vs.45.6%), not having tertiary education (75.0% vs.67.9%), living alone (16.2% vs.10.8%) and reporting poor self-rated health status (23.9% vs.16.0%).

**Table 1:** Characteristics of the respondents, according to metropolitan and nonmetropolitan areas

Characteristics	Total	Metropolitan area	Non-Metropolitan area	*P*-value
	N (%) 1074 (100%)	N (%) 526 (49.0%)	N (%) 548 (51.0%)	
Age, mean (SD)	72.51 (6.2)	72.26 (6.23)	72.74 (6.25)	
Population density	650.16	1490.04	288.06	
Gender				0.003^*^
Male	50.3%	45.6%	54.7%	
Female	49.7%	54.4%	45.3%	
Age				.578
65–74	65.2%	66.7%	63.7%	
75–84	28.7%	27.4%	29.9%	
85+	6.1%	5.9%	6.4%	
Occupational type				.276
Full-time job	10.1%	9.1%	11.1%	
Not full-time job	89.9%	90.9%	88.9%	
Education				0.010^*^
Not Tertiary degree	71.5%	67.9%	75.0%	
Tertiary degree	28.5%	32.1%	25.0%	
Marital status				.250
Unmarried	23.2%	21.7%	24.6%	
Married	76.8%	78.3%	75.4%	
Living status				0.010^*^
Alone	13.6%	10.8%	16.2%	
With others	86.4%	89.2%	83.8%	
Self-rated health status				0.001^**^
Good	80.0%	84.0%	76.1%	
Poor	20.0%	16.0%	23.9%	
BMI (kg/m2)				.328
Non-overweight	58.2%	59.7%	56.8%	
Overweight/Obese	41.8%	40.3%	43.2%	

*SD* standard deviation; min/d, minutes/day

The proportional difference in characteristics between the residents of metropolitan and nonmetropolitan areas was tested using χ2

^*^p < 0.05, ^**^p < 0.001

### Prevalence of dog ownership and dog walking status

Table 2 indicates that of the respondents, 88.0% were non-dog owner, and 12.0% owned a dog (including 8.3% of non-dog walker and 3.7% of dog walkers). The prevalence of achieving 150 min/week of leisure-time walking was 53.1%; on average, the respondents spent 218.38 min in leisure-time walking over 5.98 days/week (SD = 1.80). Additionally, the prevalence of dog ownership was 12%, and 31% of those respondents walked their dogs for an average of 232.13 min over 5.9 days/week (SD = 2.03). Chi-squared tests also revealed that older adults living in nonmetropolitan areas were more likely to be a non-dog walker (those who owned a dog but did not walk their dog) than those living in metropolitan areas (10.9% vs. 5.5%). No significant associations were identified for achieving 150 min of leisure-time walking between residents of metropolitan and nonmetropolitan areas. Mann-Whitney U test showed that older adults living in metropolitan areas were more likely to engage in leisure-time walking than were those living in nonmetropolitan areas.

**Table 2:** Dog ownership, dog-walking status, and leisure-time walking, according to metropolitan and nonmetropolitan areas

Variable	Total	Metropolitan area	Non-Metropolitan area	*P*-value
	1074	526 (49.0%)	548 (51.0%)	
% of dog ownership /dog walking category				.004^*^
Non-dog owner	88.0%	90.9%	85.2%	
Non-dog walker	8.3%	5.5%	10.9%	
Dog walker	3.7%	3.6%	3.8%	
% of dog walking in dog owners				.105
Dog owner				
- Non-dog walker	69.0%	60.4%	74.1%	
- Dog walker	31.0%	39.6%	25.9%	
% of meeting 150 min of LT walking				.053
< 150 min	46.9%	43.9%	49.8%	
150+ min	53.1%	56.1%	50.2%	
Time spent in dog walking (dog walker)				0.15
Day/Week	5.85	5.21	6.43	
Mean	232.13	182.63	276.90	
(SD) (min/week)	(211.11)	(144.57)	(252.31)	
Time spent in leisure-time walking				0.03^*^
Day/Week	5.98	5.85	6.11	
Mean	218.38	232.14	205.16	
(SD) (min/week)	(249.34)	(248.86)	(249.32)	

*SD* standard deviation, *LT* leisure time

Data are expressed as number (percentage) or mean (SD)

Difference across metropolitan area categories was tested using χ^2^ for categorical variables and Mann-Whitney U test for continuous variables

^*^p < 0.05

### Dog ownership and dog-walking status associated with 150 min of leisure-time walking in metropolitan and nonmetropolitan areas

The ORs for attaining 150 min/week leisure-time walking are presented in Table 3, according to the categories of dog ownership and dog-walking status among the residents of metropolitan and nonmetropolitan areas. Moreover, Table 3 shows that the respondents who were dog walkers living in nonmetropolitan areas were more likely to achieve ≥150 min/week of leisure-time walking according to both Model 1 (OR = 3.21; 95% CI = 1.16–8.95) and Model 2 (OR = 3.03; 95% CI = 1.05–8.77).

**Table 3:** Dog ownership and dog-walking status associated with 150 min of leisure-time walking among all respondents

Variable	Leisure-time walking (meeting 150 min/week)	
	Model 1 OR (95% CI)	Model 2 OR (95% CI)
Total		
Non dog owners	1.00 (Ref.)	1.00 (Ref.)
Non dog walkers	0.90 (0.58–1.39)	1.05 (0.67–1.65)
Dog walkers	1.86 (0.95–3.66)	1.84 (0.92–3.65)
Non-Metropolitan area		
Non dog owners	1.00 (Ref.)	1.00 (Ref.)
Non dog walkers	1.03 (0.60–1.77)	1.10 (0.63–1.93)
Dog walkers	3.21 (1.16–8.95)^*^	3.03 (1.05–8.77)^*^
Metropolitan area		
Non dog owners	1.00 (Ref.)	1.00 (Ref.)
Non dog walkers	0.79 (0.37–1.68)	0.91 (0.41–1.99)
Dog walkers	1.10 (0.43–2.80)	1.12 (0.43–2.91)

*OR* odds ratio, *CI* confidence interval

Model 1 is adjusted for gender and age

Model 2 is adjusted for gender, age, city of residence, education level, type of occupation, marital status, living status, BMI, and self-rated health status

^*^p < 0.05

## Discussion

This is the first study to provide evidence for ṭhe prevalence of dog ownership and dog walking among Taiwanese older adults, stratified by metropolitan or nonmetropolitan area. Among the respondents, the prevalence of dog ownership was 12%, and 28.8% of them walked their dogs for an average of 232.13 min over 5.9 days/week. The prevalence of dog ownership among Taiwanese older adults (12%) is slightly lower than that reported in Japan (14%) [14], the United States (15%–16%) [8, 12, 13], and the United Kingdom (22%) [11]. Moreover, the prevalence of dog walking in our sample was 31.0%, which is considerably lower than that reported in the United States (48%) and Japan (71%).

Stratification by area indicated that older adults living in nonmetropolitan areas were more likely to own a dog but less likely to walk their dog than were those living in metropolitan areas. Higher dog ownership in nonmetropolitan areas could be because older adults living in nonmetropolitan areas are more likely to live alone [21]; therefore, they are more likely to own a dog for companionship or security. Moreover, the lower prevalence of dog walking among older adults in nonmetropolitan areas could be because nonmetropolitan areas are characterized by low-density neighborhoods, and older adults are likely to live in detached single-family housing with larger yards; this provides a place for dogs to be active without needing to be walked [15, 22].

Another novel fınding of the present study highlights how metropolitan status may be a modifier between the categories of dog ownership and dog-walking status and leisure-time walking among older adults. Previous results for well-developed countries have indicated that dog walkers were more likely to meet the recommended physical activity guideline [8, 11–14]; however, our results suggest that dog-walking only appears to be more critical for nonmetropolitan older adults to engage in health-enhancing levels of recreational walking, compared with metropolitan older adults. A possible explanation for these results is that, compared with older adults living in metropolitan areas, those living in nonmetropolitan areas may have lower walkability because of poorer infrastructure (i.e., sidewalks, intersections) and less leisure options (i.e., parks, leisure facilities) in their neighborhood environment to engage in leisure-time walking [23]. Limited leisure options in the neighborhood, may facilitate the accumulation of sufficient leisure-time walking among nonmetropolitan older adults who walk their dogs regularly.

Critically, the aforementioned results indicate that promoting the initiation of dog walking among older adults who live in nonmetropolitan areas and own dogs, but who do not currently walk their dogs, could be a favorable population-based intervention strategy to achieve the recommended levels of leisure-time walking. It is also noticed that older adults living in nonmetropolitan areas were more likely to have the characteristics of living alone and reporting poor self-rated health status. Thus, effective dog-walking intervention strategies, such as changing the perceptions regarding exercise requirements of the dog or providing dog-supportive physical environments, have been suggested as modifiable points for intervention [24, 25]. These dog-walking intervention strategies targeting nonmetropolitan older adults should be considered for future physical activity intervention studies.

Several limitations should be addressed for the present study. First of all, the limited sample size for subgroup analysis may decrease the statistical power of our findings. However, the small sample size of subgroup analysis may reflect current situation of dog walking among Taiwanese older adults. Thus, future studies using prospective design with larger sample size should further investigate these associations in this context. Second, because of the cross-sectional design, we could not draw conclusions regarding the causal relationship between dog walking and the recommended levels of leisure-time walking. Third, the measurements of the present study were self-reported and thus subject to bias [16, 26]. Fourth, information of dog owners’ preference for dog breeds and dog characteristics such as dog health, age, type, or size that may be related to dog owners’ walking behavior [27], were not obtained in this study. Finally, due to the telephone-based survey, this study had a limited representative sample; therefore, sections of the population without a household telephone (approximately 7.1% in 2015) were impossible to reach [28]. Thus, the present findings may not be generalizable to the overall older adult population in Taiwan.

## Conclusion

Older Taiwanese adults living in nonmetropolitan areas who owned and walked their dogs were more likely to achieve health-enhancing levels of leisure-time walking. Thus, tailored physical activity interventions for promoting dog walking should be developed for older adults who are dog owners living in nonmetropolitan areas and who do not engage in dog walking. Future studies using qualitative design are needed as an extension from this study to find out the reasons why dog owners not walking their dogs both in metropolitan and non-metropolitan area.

## Abbreviations

BMI: Body mass index
CI: Confidence interval
IPAQ-LV: International Physical Activity Questionnaire-long version
LT: Leisure time
OR: Odds ratio
SD: Standard deviation
